# The shallow of your smile: the ethics of expressive vocal deep-fakes

**DOI:** 10.1098/rstb.2021.0083

**Published:** 2022-01-03

**Authors:** Nadia Guerouaou, Guillaume Vaiva, Jean-Julien Aucouturier

**Affiliations:** ^1^ Science and Technology of Music and Sound, IRCAM/CNRS/Sorbonne Université, Paris, France; ^2^ Lille Neuroscience and Cognition Center (LiNC), Team PSY, INSERM U-1172/CHRU Lille, France; ^3^ FEMTO-ST, UBFC/CNRS, Besançon, France; ^4^ Alta Voce SAS, Houilles, France; ^4^ Centre National de Ressource et Résilience (CN2R Lille Paris), Lille, France

**Keywords:** voice transformation, ethics, deep-fake, moral psychology, emotions

## Abstract

Rapid technological advances in artificial intelligence are creating opportunities for real-time algorithmic modulations of a person’s facial and vocal expressions, or ‘deep-fakes’. These developments raise unprecedented societal and ethical questions which, despite much recent public awareness, are still poorly understood from the point of view of moral psychology. We report here on an experimental ethics study conducted on a sample of *N* = 303 participants (predominantly young, western and educated), who evaluated the acceptability of vignettes describing potential applications of expressive voice transformation technology. We found that vocal deep-fakes were generally well accepted in the population, notably in a therapeutic context and for emotions judged otherwise difficult to control, and surprisingly, even if the user lies to their interlocutors about using them. Unlike other emerging technologies like autonomous vehicles, there was no evidence of social dilemma in which one would, for example, accept for others what they resent for themselves. The only real obstacle to the massive deployment of vocal deep-fakes appears to be situations where they are applied to a speaker without their knowing, but even the acceptability of such situations was modulated by individual differences in moral values and attitude towards science fiction.

This article is part of the theme issue ‘Voice modulation: from origin and mechanism to social impact (Part II)’.

## 1. Introduction

The human facial and vocal expressions have evolved as signals to inform and manipulate others [1,2]. By continuously modulating our facial muscles and the phonatory and articulatory structures of our vocal apparatus, we provide a rich, flexible non-verbal back-channel to our daily conversations, communicating our emotional states such as joy or surprise [3,4], our social intents such as warmth or dominance [5,6], or our epistemic attitudes, such as certainty or doubt [7,8].

**Figure 1.**
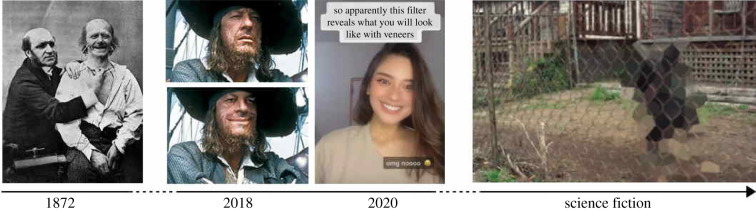
From Darwin to deep-learning: rapid technological advances in artificial intelligence create opportunities for real-time algorithmic modulations of facial and vocal expressions, which raises unprecedented societal and ethical questions. From left to right: (*a*) original studies of human facial expressions employed electric stimulation to induce muscle contraction (Guillaume Duchenne de Boulogne, reproduced in [1]); (*b*) manipulation of individual action units in still photographs using Generative Adversarial Networks (GANimation [14]); (*c*) real-time smile filters in commercial video sharing plateforms (Tiktok, ByteDance Ltd., Beijing, China); (*d*) still from the ‘Arkangel’ episode of dystopian science fiction television series *Black Mirror* (Endemol Shine UK Ltd., 2017) in which parents equip their children with anti-violence visual filters via a brain implant. Here, the device visually filters out a dog aggressively barking at the child, directly in the child’s mind. (Online version in colour.)

While our facial and vocal expressions were shaped by a long and delicate interplay of biological and cultural evolution [9,10], spectacular technological advances occurring in the past few years may soon dramatically alter how we use and experience these behaviours in daily life. Recent progress in signal processing has indeed made possible the real-time manipulation of e.g. facial expressions such as smiles [11] and vocal expressive cues such as pitch [12] or timbre [11]. Perhaps even more radically, recent advances in deep neural network architectures have provided a flexible way to learn and generate mappings (or ‘deep-fakes’ [13]) between pairs of stimuli, and opened possibilities to parametrically manipulate individual facial actions [14] (figure 1*b*) or convert one voice into several emotional variants [15]. In just a few years, combined with the unprecedented adoption of remote communication software such as video conferencing and virtual meetings, we have come to a situation where it is difficult to trust whether the smiles, laughs and frowns of our conversation partners are genuine or algorithmically modulated (figure 1*c*).

The goal of this paper is to initiate the data-driven study of expressive deep-fakes ethics (specifically here, *vocal* deep-fakes) and, inspired by the methodology of ‘experimental ethics’ [16], to quantify societal expectations about the principles that should guide their deployment.

**Figure 2.**
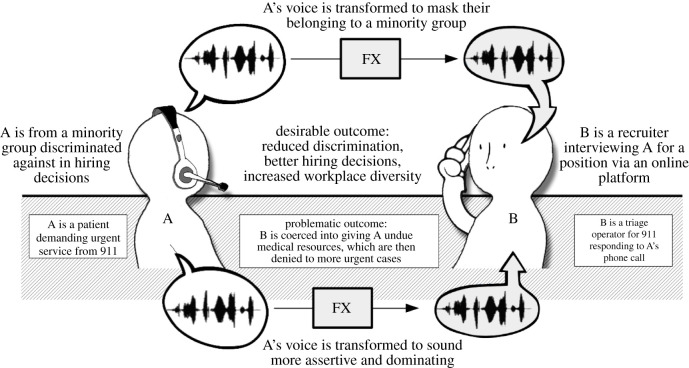
Very similar uses of voice transformation technology can lead to both desirable or problematic situations. Top: a voice transformation is used to mask the sex, accent or ethnicity of a user to eliminate discrimination in online hiring services. Situation inspired by genuine practice by the interviewing.io company [21]. Bottom: a voice transformation is used to increase the perceived dominance of a patient calling emergency medical services, who consequently gets undue medical resources from triage operators at the expense of other more urgent cases. Situation inspired by the authors’ experimental work [20].

The realistic, artificial manipulation of expressive behaviour raises unprecedented societal and ethical questions. First, it raises concerns about truthfulness. Because expressive behaviours are often thought to provide genuine cues about the sender’s emotional states [17], the ability to arbitrarily manipulate these displays opens avenues for deception: one may use, e.g. a facial filter to fake a smile despite having no intent to affiliate, or a voice transformation to appear more certain than one really is. Second, they raise concerns about fairness. Expressive behaviours in verbal interactions strongly influence subsequent behaviours. It is already known that vendors displaying positive, authentic expressions while interacting with customers sell more mobile phones [18], or that negotiators faking anger in commercial discussions obtain better prices [19]. The algorithmic manipulation of expressions designed for such situations may coerce people into making unwarranted or unfair decisions. Third, they raise concerns about autonomy. Non-verbal influences on behaviour are often non-conscious: in a study with voice transformation, mock patients calling 911 medical triage with a more dominant voice obtained more urgent medical responses from doctors but doctors did not attribute the cause of their behaviour to the patient’s voice; rather, they wrongly attributed it to more urgent medical situations [20] (figure 2, bottom). Technologies able to trigger such unconscious reactions are therefore intrinsically manipulative, as people may not be able to identify the transformation as the cause for their subsequent behaviour. Finally, they also raise concerns about transparency, as their deployment in virtual conversations lends itself to situations where a speaker does not know *how* their interlocutor is hearing or seeing them, i.e. whether a transformation of their own voice or face is applied without their knowing.

However, none of these deontological concerns warrants a straightforward moral objection to the deployment of expressive transformation technologies, because each of them also create opportunities for highly desirable situations. First, the fact that, e.g. a smiling voice transformation can be used to appear happier than one really is becomes highly desirable in the case of patients who cannot easily express emotions (e.g. amyotrophic lateral sclerosis patients who rely on assistive voice technology for communication, [22]). Second, the fact that voice or face transformations can coerce observers into subsequent actions can be desirable in interventions where people are nudged into positive behaviour [23], for instance reducing aggressive behaviour in call-centre conversations by transforming the operator’s fatigued voice [24], or applying a gender voice transformation on an online hiring platform to alleviate gender biases [21] (figure 2, top). Third, the fact that expressive transformations can be processed unconsciously may be desirable in situations where this increases their effectiveness, as seen in emotional vocal feedback [25].

Societal expectations in such situations are non-trivial and important to understand in order to inform and regulate the deployment of deep-fakes in commercial products or clinical protocols. A recently emerging methodology for doing so is that of experimental ethics, in which moral judgements about various situational vignettes are collected from relatively large samples of online participants. In recent years, this methodology has been applied to quantify societal attitudes towards new technologies such as autonomous vehicles [16] or brain stimulation [26], potential public policies such as legalizing payments to kidney donors [27], but also downright futuristic scenarios such as mind upload [28], sex robots [29] or cognitive enhancement with brain implants [30] (figure 1*d*). The experimental ethics approach allows comparing different situation variants that may make or break dilemmas (e.g. whether imagining oneself as the conductor of an autonomous car changes one’s attitude to how to react to accidents—[16]) and whether these effects are modulated by individual differences (e.g. whether a person’s familiarity with science fiction themes modulates their attitude towards robots—[29]).

Here, we employed the methodology of experimental ethics to gauge societal attitudes towards emotional voice transformation technology. We asked *N* = 303 online participants to read 24 short text vignettes describing potential applications of vocal deep-fakes, and rate how morally acceptable they thought each scenario is. Participants were presented with a cover story describing an imaginary hardware device (MyVoicePlus) able to transform the emotional quality of a voice in real-time, which was said to be currently considered by a startup company for commercial deployment in various situations. The vignettes describing potential applications of the device varied among four factors:

(i) whether the user of the device was the participant or an unknown other;
(ii) whether the transformations were used therapeutically, or to enhance user capacities;
(iii) whether the transformations operated on positive (enhancing smiling) or negative emotional expressions (reducing anxiety, reducing anger);
(iv) and whether the transformation affected how the user’s voice is heard by others (i.e. the user’s production), how the user hears other persons’ voices (i.e. the user’s perception), or whether it is used in a situation where the user hears their own manipulated voice (i.e. feedback).

For each vignette, participants first rated the acceptability of the situation, and were then presented with two potential dilemmas involving lying about the true purpose of the transformation in order to improve its effectiveness. Finally, for all of these judgements, we examined associations with individual differences in participants’ attitudes towards morality (Moral Foundations Questionnaire, MFQ [31], measuring factors of harm–care, fairness–cheating, loyalty–betrayal, authority–subversion and purity–degradation) and toward technology and science fiction (Science Fiction Hobbyism Scale, SFH; [28]), two factors found relevant in previous research about the moral reception of new technologies [26,28,29,32] (see §4 for details of the procedure).

Although our study is exploratory and we did not preregister any formal hypotheses, a number of loose predictions can be made from the literature about how our variables of interest impact participants’ moral judgements. First, similar experiments with emerging technologies such as autonomous vehicles [16] or brain stimulation [26] have documented situations of social dilemma, in which participants accept things for themselves (i.e. a car that favours its driver, rather than pedestrians) that they would otherwise reject for others. Second, across diverse forms of enhancement (e.g. memory, general intelligence, mood, etc.), participants are widely reported to be more comfortable with technologies that enhance capacities towards the norm (i.e. that are used therapeutically) than above the norm [30,33]. Finally, to the best of our knowledge, there is no straightforward equivalent in the literature of whether, e.g. manipulating positive or negative emotions, or manipulating a user’s perception or production, has any impact on a participant’s judgement of acceptability. Whether participants feel more comfortable with, e.g. smiling or anxiety filters, and filters that affect their produced voice or their perception of how others sound, is an open non-trivial question [34], which our study wishes to address.

## 2. Results

### (a) Acceptability of overtly using the technology

We first evaluated how morally acceptable our participants (*N* = 303) thought the use of a voice transformation device was, when the true purpose of the technology was overtly known to all involved parties.

#### (i) Voice transformations are in general well accepted in the population

Across situations, the moral acceptability of overt vocal transformation was strongly significantly higher than neutral (*M* = 6.49 > 5; one-sample *t*-test against mid-point, averaging all acceptance scores across vignettes: *t*(302) = 146, *p* < 0.001).

**Figure 3.**
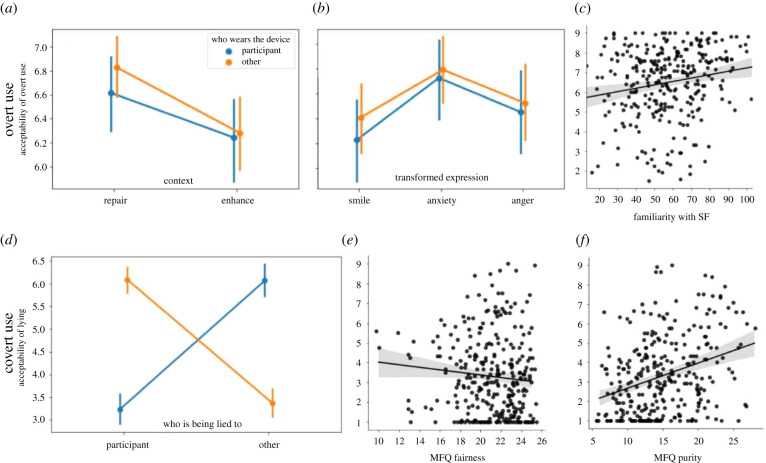
Moral judgements of overt and covert use of voice transformations. Top row: overt use. (*a*) The moral acceptability of overt vocal transformation was higher than the neutral midpoint, and therapeutic transformations even more so than transformations used to enhance user capacities. (*b*) Situations in which transformations aimed at weakening the two negative emotions of anxiety or anger were better accepted than situations in which transformations aimed to enhance smiling. (*c*) Across situations, acceptability was positively associated with the participants’ familiarity with science fiction. Bottom row: covert use involving lying about the true purpose of the transformation in order to improve its effectiveness. (*d*) Participants considered it morally acceptable that the user of the transformation hides its true purpose to others but hiding the transformation to the person using the device was totally unacceptable. (*e*,*f*) The acceptability of lying to the person using the device was negatively associated with the participants’ concern with fairness, and positively with purity. (*a*,*b*,*d*) Across conditions, there was no effect of whether the user of the device was the participant or an unknown other. Error bars: 95% confidence intervals. (Online version in colour.)

Because of heteroscedasticity (Breush-Pagan: *F*(6, 296) = 3.23, *p* = 0.004), we tested the effect of individual characteristics on this judgement with multiple iterated re-weighted least squares (IRLS) regression (Huber weights, HC3 correction). Acceptability was significantly positively associated with the participants’ familiarity with science fiction (*β* = 0.014, *z* = 2.75, *p* = 0.006; figure 3*c*) and marginally positively associated to participant’s reliance on MFQ purity (PU) (*β* = 0.04, *z* = 1.85, *p* = 0.064). No other MFQ factors regressed significantly (all  ps>0.1). The marginal positive association with MFQ PU differed from other studies of similar technologies where purity was found negatively correlated with acceptability (e.g. mind upload [28]; sex robots [30]).

#### (ii) A therapeutic context makes them even more acceptable

We tested the effect of the goal to repair or enhance on situation acceptability by averaging within-participant scores for overt acceptability over therapeutic (*n* = 6 vignettes) and enhancing situations (*n* = 6 vignettes), and testing for population differences with a one-way repeated-measure ANOVA. Repair–enhance had a significant main effect on situation acceptability (*F*(1, 302) = 47, *p* < 0.001), with therapeutic situations (*M* = 6.7) being (even) more acceptable than enhancing situations (*M* = 6.2; figure 3*a*).

To test whether the effect of repair or enhance was associated with individual characteristics, we computed the within-participant difference between acceptability scores averaged over both types of vignettes, and computed multiple ordinary least-square (OLS) regression (Breusch–Pagan heteroscedasticity test: *F*(6, 296) = 0.39, *p* = 0.88). The better acceptability of repair situations was not significantly associated with individual differences in MFQ or science fiction familiarity (*R*^2^ = 0.008, *F*(6, 296) = 0.38, *p* = 0.89).

#### (iii) Manipulating perception is less acceptable than manipulating production

Similarly, we tested the effect of whether situations described voice transformation as affecting how the user’s voice is heard by others (condition *production*: *n* = 4 vignettes), how the user hears other persons’ voices (condition *perception*: *n* = 4 vignettes), or whether the user hears their own manipulated voice (condition *feedback*: *n* = 4 vignettes) by averaging acceptability scores within-participant over the three types of vignettes and testing for population differences with a one-way repeated-measure ANOVA. There was a significant effect of the production–perception–feedback variable (*F*(2, 604) = 7.5, *p* = 0.001), with transformations affecting the user’s production being more acceptable than perception and feedback. Both latter conditions share the fact that the device manipulates what the participant hears, regardless of whether it is the participant’s own voice or that of another person.

We tested for associations with individual characteristics by computing the within-participant pairwise differences between acceptability scores averaged over all three types of vignettes, and computing multiple OLS regression (Breusch–Pagan heteroscedasticity test: perception–production *F*(6, 296) = 0.27, *p* = 0.96; feedback–production *F*(6, 296) = 0.66, *p* = 0.6815). The difference of acceptability between these situations was not associated with participant MFQ or SFH (perception–production: *R*^2^ = 0.006, *F*(6, 296) = 0.31, *p* = 0.93; feedback–production: *R*^2^ = 0.011, *F*(6, 296) = 0.571, *p* = 0.75).

### (b) Acceptability of covert uses

For each situation, we then tested the acceptability of lying about the true purpose of the device, in order to increase the transformation’s effectiveness, in two situations which either involved the user’s lying to their interlocutors, or the device’s prescriber’s lying to the user themselves. Because using the transformation overtly was generally well accepted (see above), and because we presented situations in a context where lying about the transformations would also improve their effectiveness (see §4), these situations can be regarded as genuine moral dilemmas in which the deontologically blamable act of lying is balanced by the utilitarian value of the resulting improvement of performance.

#### (i) Using the transformation covertly is not a problem…

Although more acceptable situations were more acceptable to lie about (OLS regression over vignettes averaged between-participants: *R*^2^ = 0.61, *F*(1, 22) = 34.61, *p* < 0.001; Breusch–Pagan heteroscedasticity test: *F*(1, 22) = 0.13, *p* = 0.72), lying was generally regarded as non acceptable by our participants (*M* = 4.69 < 5, *t*(302) = 3.19, *p* = 0.001).

However, there was a very large interaction with which person is being lied to (one-way rm-ANOVA: *F*(1, 302) = 631, *p* < 0.001; figure 3*d*): somewhat surprisingly, participants considered it morally acceptable that the user of this device hides its true purpose to others (*M* = 6.08 [5.87, 6.3]; one-sample *t*-test against mid-point: *t*(302) = 9.99, *p* < 0.001).

Because of marginal heteroscedasticity (Breusch–Pagan: *F*(6, 296) = 1.72, *p* = 0.11), we tested the association of the acceptability of users’ lying to others with individual characteristics with multiple IRLS regression. The acceptability of lying to others was not found associated with any of the MFQ subscales (best, PU: *β* = 0.033, *z* = 1.33, *p* = 0.18), but was positively influenced by science fiction familiarity (*β* = 0.01, *z* = 1.95, *p* = 0.05).

#### (ii) …unless it is hidden from the user of the device

However, hiding the transformation to the person using the device appeared totally unacceptable (*M* = 3.3 < <5, one-sample *t*-test against mid-point: *t*(302) = −14.9, *p* < 0.001), even though the transformation was presented as more effective for the user when doing so (figure 3*d*).

As above, we tested the association of the acceptability of lying to the device’s user with individual characteristics, using multiple IRLS regression (Breusch–Pagan heteroscedasticity test: *F*(6, 296) = 1.28, *p* = 0.26). The low acceptability of lying to the user was driven (i.e. negatively associated) by participants high on the MFQ subscale of fairness (*β* = −0.1395; *z* = −3.461, *p* = 0.001; figure 3*e*) but attenuated (i.e. positively associated) for participants high on MFQ purity (*β* = 0.0930, *z* = 3.65, *p* < 0.001; figure 3*f*) and loyalty (*β* = 0.09, *z* = 2.71, *p* = 0.007). The acceptability of lying to the user was also associated with science fiction familiarity (*β* = 0.014, *z* = 2.38, *p* = 0.017), with greater familiarity making lying to the user more acceptable.

### (c) Acceptability of voice transformations is not influenced by seeking self profits

To test for the effect of either depicting situations where the user was the participant or an unknown person, we conducted a mixed ANOVA with self–other as a between-participant factor, and vignette conditions (repair–enhance, positive–negative transformation, production–perception–feedback) as within-participant factors. There was no statistical difference of acceptability between overt situations which depicted the participant as the user benefiting of the device (*M* = 6.43), and situations where the user was an unknown person (*M* = 6.55; no main effect, *F*(1, 301) = 0.37, *p* = 0.54). Neither did the effect of self–other interact with any of the other variables: regardless of whether the user was themselves or others, participants thought similarly of differences between situations meant to repair and enhance (no interaction self–other × repair–enhance: *F*(1, 301) = 1.68, *p* = 0.20; figure 3*a*), of differences between situations involving smiling, anger or anxiety (no interaction self–other × transformation: *F*(1, 301) = 1.43, *p* = 0.23; figure 3*b*), and of differences between devices affecting the user’s production, perception or feedback (no interaction self–other × production–perception–feedback: *F*(2, 602) = 0.047, *p* = 0.95).

Similarly, participants did not judge less acceptable the covert situations where the true purpose of the device was hidden from them (regardless of whether they were its user, or not), compared to situations where it was hidden from unknown others (rm-ANOVA, with concealed participant–other as within-participant factor, *F*(1, 302) = 0.0026, *p* = 0.87; figure 3*d*). In other words, the relatively high acceptability of users’ lying to others did not depend on whether the participant was the user of the device or the person whom the transformation is hidden from; and the low acceptability of lying to the device’s users did not depend either on whether the user was the participant themselves or an unknown other.

In sum, contrary to situations like pedestrian dilemmas in autonomous vehicles [16], there was radically no evidence of social dilemma regarding the use of voice transformations, in which one would e.g. accept for themselves what they would blame others for, even in situations involving the blamable act of lying.

### (d) The nature of the emotion impacts the moral acceptability of the transformation

Finally, we tested the impact of what emotion is transformed on the acceptability of the situation, as well as the interaction with the repair–enhance factor. We averaged within-participant scores of overt acceptability over repair–enhance situations concerning anxiety (*n* = 4 vignettes; repair: 2), anger (*n* = 2 vignettes; repair: 1) and smile vignettes (*n* = 6; repair: 3), and tested for population differences with a two-way rm-ANOVA.

There was a main effect of emotion: situations in which transformations aimed at weakening the two negative emotions of anxiety (*M* = 6.8) or anger (*M* = 6.5) were better accepted than situations involving transformations enhancing smile (*F*(2, 604) = 24.47, *p* < 0.001), although the latter remained well accepted at *M* = 6.3 (figure 3*b*).

The effect of emotion also interacted significantly with the repair–enhance factor (*F*(2, 604) = 21.3, *p* < 0.001), with transformations aiming to weaken negative emotions benefiting more of the therapeutic condition (Δ = +0.56) than the transformation targeting positive emotions (Δ = +0.35). The effect was maximal for the repair of anxiety (repair: *M* = 7.17; enhance: *M* = 6.34).

Similarly, in covert situations, it was more more acceptable to hide the purpose of a transformation aiming to weaken negative emotions than a transformation aiming to enhance smile (one-way rm-ANOVA; main effect of transformation: *F*(2, 604) = 8.3, *p* < 0.001).

Finally, we tested whether these differences between positive and negative transformations were associated with individual differences, by computing the within-participant pairwise differences between acceptability scores averaged over all three types of transformations, and computing multiple OLS regression (Breusch–Pagan heteroskedasticity test: anxiety–smile *F*(6, 296) = 0.59, *p* = 0.74; anger–smile *F*(6, 296) = 0.73, *p* = 0.62). The difference of acceptability between these situations was not associated with participant MFQ or SFH (anxiety–smile: *R*^2^ = 0.031, *F*(6, 296) = 1.58, *p* = 0.15; anger–smile: *R*^2^ = 0.023, *F*(6, 296) = 1.137, *p* = 0.34).

## 3. Discussion

We reported here on an experimental ethics study in which *N* = 303 online participants evaluated the acceptability of vignettes describing potential applications of expressive voice transformation technology. We found that vocal deep-fakes were generally well accepted, notably in a therapeutic (versus enhancement) context; when they corrected negative emotions rather than enhanced positive emotions; and when they manipulated a speaker’s production rather than perception. Surprisingly, transformations remained well-accepted even when the user lied to their interlocutors about using them and, unlike other emerging technologies such as autonomous vehicles, there was no evidence of social dilemma in which one would accept for others what they resent for themselves. The only real moral objection to vocal transformations appeared related to situations in which they were applied to a speaker without their knowing, with the acceptability of such situations being modulated by individual differences in moral values and attitude towards science fiction.

The fact that voice transformations are generally well-accepted, with average scores across situations well above the scale mid-point, first and foremost shows that the western, young, educated population studied here is sympathetic to the idea of customizing one’s own emotional expression with technology, when these technologies become available. This attitude, at least for the range of scenarios tested here, seems consistent with transhumanistic views for which technology should be used to enhance human capacities and improve happiness [35] as well as control for emotional or neurological limitations (e.g. taking anti-love drugs to curb affect in divorce situations [36]).

Contrary to other moral psychology studies where individual attitudes to MFQ purity negatively correlated with acceptability of cognitive enhancement or mind upload [28], acceptability here was facilitated by the participants’ reliance on the purity dimension. This may suggest that voice transformations are not seen as a breech of human integrity, but rather as a way to improve control and self-determinacy (i.e. an *anthropotechnical tool* for self-customization [37]). In a contemporary society promoting continuous self improvement, the good reception of this kind of technology is thus perhaps not surprising [38]. However, it should be noted that the MFQ purity construct has come under recent debate (e.g. it may be interpreted differently by religious and non-religious individuals [39]), and further research is needed to ascertain what this construct measures in our specific sample of participants.

The good general acceptance of voice transformations was further improved in therapeutic situations, which were judged more acceptable that situations merely aiming to enhance user capacities [40]. This attitude is consistent with what is reported in other empirical studies of cognitive enhancement [26,30,33], and with imperatives put forward by the bioethics literature [41,42]. It confirms that the therapy-enhancement distinction is morally salient to the public concerning potential display of expressive voice transformation technology.

Acceptance was also higher for situations which manipulated the production of an expression than situations which manipulated its perception. That participants should be biased against the latter somehow contradicts the expectation that covert changes that are internal to the individual would have less broad impact on others than changes affecting their outward expression [43]. This preference may reflect a worry about having one’s real experience distorted, as one could worry e.g. about mood-enhancer drugs such as SSRIs altering one’s sense of living truly (*is it me or the Prozac enjoying this?* [40,44]), even though in the case of Prozac these bioethical concerns do not seem shared by the general population [45]. Since the production and perception situations could be compared respectively with the use of Instagram filters (which are now common; figure 1*c*) over augmented-reality (AR) glasses (which are not yet), it would be interesting to follow up on these results in the next few months, as several announced AR devices such as Apple Glasses may gain popularity and modify these attitudes ([46]; see also below about science fiction familiarity).

In a second set of questions, we collected judgements about concealed-use situations, and presented them in a context where lying about the transformations would also improve their effectiveness (see §4 *Judge how acceptable it is to lie to your entourage […], knowing that this would improve the effectiveness of the device*). The fact that voice transformations are generally thought desirable in ‘overt’ situations makes these ‘covert’ situations appear as genuine moral dilemmas, in which the deontological imperative against lying is balanced against the utilitarian benefits of self-improvement. For these situations, both sides of the debate were clearly reflected in participant judgements: on the one hand, acceptance of lying was negatively associated with MFQ fairness; on the other hand, as was the case in overt situations, acceptance scores for these situations were also positively associated with MFQ purity, which attenuated the generally low acceptability of covert use.

Strikingly though, in all of these dilemma as well as in the less problematic ‘overt‘ situations, we found radically no evidence of a social dilemma where a participant would refuse for themselves what they think acceptable for others. This held whether participants envisioned modifying their own voice, or that of others; and whether participants were being lied to regarding their perception, or whether they lied to others. This absence of effect of who benefits from the device when judging its acceptability is in stark contrast with typical sacrificial scenarios (like the trolley problem or, more recently, pedestrian versus driver dilemma in autonomous vehicles), in which participants tend to value self-preservation [16,47]. This suggests that participants judge voice-transformation technology primarily with a utilitarian perspective, treating the welfare of everyone as of equal importance, ‘from the point of view of the universe’ [48] regardless of whether they are near or far, our children and friends or absolute strangers, human or animal [43]. While this does not mean that self-preservation biases could not be created, for instance for situations involving finite supply [26] or larger individual cost [16], the fact that voice transformation should be judged so impartially suggests that there currently is no social obstacle to the massive deployment of such technologies in (here, western) societies.

Even though there was no effect of self–other, covert dilemma was very strongly biased against lying to the person wearing the prosthesis (i.e. regardless of who that person was: self or other). This attitude may be an effect of describing the device in our cover story as a physical prosthesis, for which ‘installing’ it covertly would be seen as an unacceptable breach of consent–autonomy [49]. To control for physicality, future work could e.g. extend this study to assess the acceptability of software effects (filters) deployed in virtual meeting software.

Unexpectedly, transformations aiming to enhance positive expressions (smiles) were judged less acceptable than those aiming to reduce negative expressions (anxiety, anger). This asymmetric pattern of result contrasts with a purely hedonic view, in which making people ‘feel as good as possible, and feel least bad’ [50] would be equally valued. Rather, it may indicate that curbing negative expressions is valued less for the gain of valence than for an Aristotelician inclination for control over oneself, because negative emotions such as portrayed here (stress, anxiety, fear) are viewed as less deliberate and more automatic than smiling [51]. This view is also consistent with our interpretation of MFQ purity as valuing self-determinacy. If true, this may prefigure a situation where, when broadly adopted, expressive technology would shift the moral responsibility associated to certain emotions or behaviours: expressions which were once normal to not control (e.g. one cannot be blamed for stress [51]) may become controllable, and thus blamable and subjected to social demand (e.g. *‘why didn’t you put stress-control on?’*, [52]). To further test this idea, it would be interesting to examine scenarios involving non-deliberate positive expressions (eg. using a transformation to avoid giggling uncontrollably at a funeral) or to examine how the present results are modulated by cultural differences in emotional display norms [53].

Finally, across-the-board positive associations with the participants’ familiarity with science fiction indicate a robust effect of cultural conditioning on the acceptance of voice transformation technology. As already remarked for brain implants [28] or cognitive enhancement [30], exposure to futuristic themes and ideas appears associated with less resistance to technologies which challenge our conception of human nature. The influence of science fiction themes is already well studied as a source of inspiration for real-world technological innovation, e.g. in space [54] or nanotechnologies [55], but it appears that it also plays a role in the reception of new technology by the general public [56]. One consequence is that the attitude towards voice transformations may co-vary with cultural differences in attitudes towards new technology (e.g. robots in Japan [57]).

One obvious limitation of this work is our focus on a sample of predominantly young and educated western participants (i.e. college students), which is representative neither of the generation population in western countries (as would survey pools constructed to match the composition of a given adult population by gender, age, education and ethnicity—[27]), nor of the more global non-WEIRD population [58]. Although research suggests that instruments such as the MFQ are relatively stable across cultures [59], there is an emerging corpus of work attempting to diversify moral psychology research samples [60,61], and to conduct cross-cultural comparisons with massive online methodologies [62]. Such initiatives will be particularly needed when evaluating the acceptability of information technologies such as deep-fakes, which are spreading equally fast in western and non-western countries [63].

The use of vignettes in experimental ethics approaches also comes with several limitations. First, the intensity of reactions elicited by the stories may be limited by the immersion of the participant, or the vividness of their imagination [30], and reading a vignette, especially one describing an intense emotional situation, may not elicit reactions as strong as in the corresponding real-life situations [64]. Here, we moderate these limitations by including an elaborate cover story presenting the device as being considered for commercialization by an actual voice technology company, and stating that participant responses will have weight in future commercial decisions. Second, all of these scenarios consider idealized transformations which are assumed to be non-identifiable as fake, and properly recognized as their intended emotion. As these technologies soon become available, future work could consider measuring reactions to more tangible situations (e.g. upon hearing one’s own voice modified by the device), studying situations in which voice transformations are not recognized as genuine behaviour (e.g. how comfortable am I to use a filter that may sound robotic at times?), or combining the approach with qualitative ethnographic methods documenting the appropriation of the device by potential users (e.g. how real call-centre operators end up using a smile transformation) [65]. Finally, it should also be noted that, even though we designed the present 12 vignettes to span a wide range of situations, it remains an open question whether our conclusions generalize to other types of vocal deep-fakes, and/or other types of situations than those tested here.

Feelings and emotions are at the forefront of the political behaviour of citizens and policy makers [66]. It will be essential for our societal future to clarify the determinants of moral judgements about technologies able to customize and control these behaviours, in order to guide norm-setting regarding their applications.

## 4. Material and methods

### (a) Participants

*N* = 303 participants (*M* = 25.7; female: 156) took part in an online study, administered via a Qualtrics questionnaire (Qualtrics International Inc., Seattle, WA). All were French residents, recruited by the INSEAD-Sorbonne Université Behavioural Laboratory among a population consisting mainly of university students. Of participants, 213 (70.3%) had completed at least a Bachelor’s degree, and 116 (38%) had at least a Master’s degree. Participants were randomised into one of two self–other conditions. For each condition, participants were presented 12 vignettes of scenarios assessing three within-participant factors tested for their possible impact on moral acceptability (see §4c). For each vignette, participants answered three questions about their perceived moral acceptability of the situation (see §4d), which creates a total of 36 answers for each participant.

### (b) Procedure

Participants were initially presented a cover story describing an imaginary hardware device able to transform the emotional quality of a voice in real-time, both in the user’s voice (for others to hear) and in the user’s ear (i.e. transforming the emotions of others’ voices). The device, named ‘MyVoicePlus’, was presented as being considered for possible commercial and/or clinical deployment by a French startup company. The cover story included mock photographs of the device (consisting of both an in-ear prosthesis and a larynx piece, disguised as jewelry), as well as references to technical voice-transformation literature (e.g. [67]) allegedly describing the algorithms implemented in the device (see electronic supplementary material). Participants were told that the startup was commissioning the study to evaluate the societal acceptability of their technology in various usage scenarios, and that their collective judgements would condition the deployment of the technology.

After reading the cover story, participants were presented a series of *n* = 12 short situational vignettes, each describing a potential application of the voice-transformation device (see following section). There were two between-participant conditions, in which participants either read vignettes that described the participant as the user of the device (condition *self*; *N* = 150), or vignettes describing otherwise-identical situations in which the device was applied to others and in which participants were in the position of the user’s conversation partners (condition *other*; *N* = 153). In each self–other condition, vignettes included a number of within-participant conditions, which we describe below. For each vignette, participants were asked to answer three questions about how morally acceptable they think the situation was (see §4d).

Finally, after completing the questions for all vignettes, participants were asked to complete two standard questionnaires measuring attitudes towards morality (Moral Foundations Questionnaire MFQ; [31]) and toward technology and science fiction (Science Fiction Hobbyism Scale; [28]). The study lasted on average 30 min.

### (c) Vignettes

We created *n* = 12 short text vignettes describing potential applications of the voice-transformation device in concrete daily life situations. Vignettes varied among three situational factors, which were encoded as within-participant variables to test for their impact on the acceptability of the device:

(i) whether voice transformations are used to repair (e.g. therapeutically) or enhance user capacities (condition *repair*: *n* = 6; *enhance*: *n* = 6). Examples of *repair* vignettes included using the device to help a depressive patient communicate with their close ones with a more enthusiastic tone of voice; examples of *enhance* situations included using the same transformation to help a politician gather more following. In *repair* vignettes, the device was described as being prescribed to the user by a doctor; in *enhance* vignettes, the device was recommended by a life coach.
(ii) the kind of voice transformation operated by the device, either reducing *anger* (*n* = 2; e.g. making angry customers’ voices less taxing to attend to, for call-centre operators), reducing *anxiety* (*n* = 4; e.g. helping a budding actor overcome stage-fright) or enhancing *smile* (*n* = 6; e.g. helping a waiter gather more tips from customers).
(iii) whether the voice transformation affects how the user’s voice is heard by others (condition *production*: *n* = 4), how the user hears other persons’ voices (condition *perception*: *n* = 4), or whether it is used in a situation where the user hears their own manipulated voice (condition *feedback*: *n* = 4). Examples of the feedback condition include having a post-traumatic stress disorder (PTSD) patient listen to their own voice made less anxious as they retell their traumatic event [68].

All 12 vignettes were written in two matched versions, in which the user of the device was either the participant (e.g. *imagine you are a depressive patient, and your doctor is advising you to use a voice-transformation device…*; condition *self* : *n* = 12) or an unknown other (e.g. *A depressive patient…; condition *other**: *n* = 12. Condition *self–other* was randomly assigned between-participant; all other conditions were varied within-participant, in random order. All vignettes are available with English translation in the electronic supplemental material.

### (d) Measures

After reading each vignette, participants answered three questions about:

(i) how morally acceptable they think the situation is (*Judge how morally acceptable it is to use the MyVoicePlus device in such a situation’; FR: A quel point jugez-vous cette utilisation du produit MyVoicePlus™ moralement acceptable?*)
(ii) how morally acceptable they think it would be for the user to use the device covertly, i.e. to lie to their conversation partners that they are either talking to them, or hearing them, with a modified voice, knowing that this may improve the effectiveness of the device by up to 70%. (*Judge how acceptable it is to lie to your entourage about using the voice transformation, knowing that this would improve the effectiveness of the device’; FR: ‘A quel point jugez-vous acceptable le fait de cacher -votre entourage l’existence de la transformation de voix, en sachant que cela augmente considérablement l’efficacité du dispositif?*)
(iii) how morally acceptable they think it would be to hide the true purpose of the device from its own user, i.e. that the users themselves do not know that they are either talking, or hearing others, with a modified voice. (*Judge how acceptable it is that the [doctor/coach] should lie to the user about the voice transformation, knowing that this would improve the effectiveness of the device’; FR: A quel point jugez-vous acceptable le fait que le médecin vous cache l’existence de la transformation de voix, en sachant que cela augmente considérablement son efficacité?*)

Answers to all three questions were rated using a 9-point Likert scale, anchored by 1 *totally unacceptable* and 9 *totally acceptable*.

### (e) Attitude questionnaires

In addition to providing moral judgements about the vignettes, participants completed two questionnaires measuring their attitudes toward morality (Moral Foundations Questionnaire MFQ; [31]) and toward technology and science fiction (Science Fiction Hobbyism Scale SFH; [28]).

The MFQ consists of 32 short questions (30 items + 2 foil items) about how relevant various considerations are (e.g. *whether or not someone suffered emotionally*) when deciding whether something is right or wrong, rated from 1 (*not at all relevant*) to 7 (*extremely relevant*), and how much the participant agrees with various moral positions (e.g. *compassion for those who are suffering is the most crucial virtue*; rated from 1 (*strongly disagree*) to 7 (*strongly agree*). In accordance with typical MFQ analysis [31], we grouped and averaged each participant responses along the five subscales of care–harm (6 items; e.g. *whether or not someone suffered emotionally*), fairness–cheating (5 items; e.g. *whether or not some people were treated differently from others*), loyalty–betrayal (6 items; e.g. *whether or not someone did something to betray their group*), authority–subversion (5 items; e.g. *whether or not an action caused chaos or disorder*), and purity–degradation (6 items; e.g. *whether or not someone violated standards of purity and decency*). None of the items were reverse-coded. In this work, we used the back-translated French-language version of the MFQ designed by Métayer & Pahlavan [69]. A previous study [69] validated MFQ-French on a sample of similar demographics as the present study and found it had acceptable internal validity (*N* = 538 participants; care: Cronbach *α* = 0.64; fairness: *α* = 0.67; loyalty: *α* = 0.65; authority: *α* = 0.73; purity: *α* = 0.79) and one-month test-retest validity (*N* = 40; care: r=0.53, 95% CI [0.26,0.72]; fairness: r=0.66,[0.43,0.80]; loyalty: r=0.66,[0.44,0.81]; authority: r=0.75, [0.57, 0.86], purity: r=0.88,[0.78,0.94]; all  ps<0.01). The fit to a 5-factor structure, while significantly better than an alternative 3-factor model, was comparably poorer (*N* = 538; Comparative Fit Index, CFI: 0.82; root mean squared error of approximation, RMSEA: 0.065), a known issue common to the American version and discussed elsewhere [70,71].

In accordance with recommendations of Métayer & Pahlavan [69] and compared to the American version, two MFQ items (fairness: *I think it’s morally wrong that rich children inherit a lot of money while poor children inherit nothing’*; authority: *‘Men and women each have different roles to play in society*) were removed from the French translation to improve internal consistency. In our data (*N* = 303), the internal validity of the 5 MFQ constructs was comparable to the sample of Métayer & Pahlavan [69] for purity–degradation (*α* = 0.74, [0.70, 0.78]), fairness–cheating (*α* = 0.65, [0.58, 0.70]), loyalty–betrayal (*α* = 0.63, [0.56, 0.69]) and authority–subversion (*α* = 0.68, [0.62, 0.73]), but was poor for the care–harm construct (*α* = 0.55, 95% CI [0.45, 0.61]). Confirmatory factor analysis for the 5-factor model was significant (GLS fit, *χ*^2^(340) = 575, *p* < 0.001), fitting data adequately on some measures (RMSEA = 0.048) but relatively poorly on others (CFI = 0.48). We opt to conform to the recommendations of a more extensive validation (with a sample size nearly twice as big as our current sample [69]) and use the 5-factor model for our current analysis. However, the present data adds evidence to the fact that, as already discussed elsewhere [71], significant elements of the MFQ covariance structure are not captured by this model.

The SFH scale [28] consists of 12 items and measures individuals’ cultural exposure to futuristic technology and science fiction themes (examples of items: *I often think about what machines are like in the future*, *I often spot science or technology related errors in science fiction films, TV series, or books’*). All items are rated from 1 (*strongly disagree*) to 7 (*strongly agree*), with higher scores indicating higher science fiction familiarity. None of the items were reverse-coded. A previous study [29] validated the scale on *N* = 172 participants and found it had good psychometric properties (all factor loadings > 0.57; Cronbach’s *α* = 0.92). In this work, we used our own, non-validated French-language translation of the SFH. In our data (*N* = 303), the internal validity of the SF construct was also good (*α* = 0.89, [0.888, 0.898]).

### (f) Statistical analyses

There were two dependent variables (DVs) in the study, measuring the acceptability of overt (DV1) and covert (DV2) use of voice transformations. DV2 was constructed by recoding the two questions about concealed use (lying to the user, and lying to others) as a single DV measured in two conditions (who is being lied to).

The study’s vignettes spanned a number of situation characteristics, each described as a combination of independent variables (IVs). There was one between-participant IV (self–other), three within-participant IVs for DV1 (repair–enhance, smile–anxiety–anger, production–perception–feedback) and an additional two within-participant IVs for covert DV2 (lying to user–other, and lying to participant–other). We analysed the effect of IVs on both DVs using one-way, repeated-measures or mixed ANOVAs, by averaging acceptability scores within-participant over the vignettes corresponding to each condition tested.

In addition, there were six measures of individual characteristics (MFQ: 5 constructs; SFH: 1 construct). We tested the association of these individual characteristics with the study’s DVs by computing within-participant averages of acceptability scores (one data point per participant) and multiple regression. We tested for residual–prediction heteroscedasticity with the Breusch–Pagan test. In case of homoscedasticity, we used multiple ordinary least square (OLS) regression; in case of heteroscedasticity, we used iterated re-weighted least-square (IRLS) regression with Huber weighting and HC3 correction. All analyses were conducted in Python (3.6.8), using the pingouin (0.3.12) and statsmodels (0.12.2) packages.

## Data accessibility

Experimental data and analysis code (open-source, Python) are available as electronic supplementary material at https://github.com/creamlab/deep-ethics.
